# Investigation of siRNA Nanoparticle Formation Using Mono-Cationic Detergents and Its Use in Gene Silencing in Human HeLa Cells

**DOI:** 10.3390/cancers5041413

**Published:** 2013-11-01

**Authors:** Yuma Yamada, Ryosuke Suzuki, Hideyoshi Harashima

**Affiliations:** Laboratory for Molecular Design of Pharmaceutics, Faculty of Pharmaceutical Sciences, Hokkaido University, Kita-12, Nishi-6, Kita-ku, Sapporo 060-0812, Japan; E-Mails: u-ma@pharm.hokudai.ac.jp (Y.Y.); ryosukes@sci.hokudai.ac.jp (R.S.)

**Keywords:** mono-cationic detergent, gene silencing, siRNA delivery, nanoparticle, multifunctional envelope-type nano device (MEND)

## Abstract

The focus of recent research has been on the development of siRNA vectors to achieve an innovative gene therapy. Most of the conventional vectors are siRNA nanoparticles complexed with cationic polymers and liposomes, making it difficult to release siRNA. In this study, we report on the use of MCD, a quaternary ammonium salt detergent containing a long aliphatic chain (L-chain) as an siRNA complexation agent using human HeLa cells (a model cancer cell). We prepared siRNA nanoparticles using various MCDs, and measured the diameters and zeta-potentials of the particles. The use of an MCD with a long L-chain resulted in the formation of a positively charged nanoparticle. In contrast, a negatively charged nanoparticle was formed when a MCD with a short L-chain was used. We next evaluated the gene silencing efficiency of the nanoparticles using HeLa cells expressing the luciferase protein. The results showed that the siRNA/MCD nanoparticles showed a higher gene silencing efficiency than Lipofectamine 2000. We also found that the efficiency of gene silencing is a function of the length of the alkyl chain in MCD and zeta-potential of the siRNA/MCD nanoparticles. Such information provides another viewpoint for designing siRNA vectors.

## 1. Introduction

To date, many siRNA vectors for *in vitro* and *in vivo* use have been reported, and most are comprised of siRNA nanoparticles complexed with cationic polymers and liposomes [1,2,3,4,5,6,7]. Since it is known that the physicochemical properties of nanoparticles affect their bioactivities, the formation of a nanoparticle that contains nucleic acids and polycations has been a subject of active investigations [1,2,3,8,9,10,11,12,13,14]. A considerable body of information is available concerning pDNA [8,9,10,11,12,13,14]. It has been reported that an important process for efficient transgene expression is the intranuclear disposition of pDNA rather than its delivery to the nucleus [15,16]. We previously reported that, for condensed DNA particles, a close relationship exists between the efficiency of DNA release and transfection activity, when biocleavable polyrotaxanes (DMAE-SS-PRX) are used, in which the cationic density can be easily controlled [17]. We also indicated that a very high efficiency of DNA release has a positive influence on transcription, but that it would inhibit the post-transcription process; nuclear mRNA export, translation and related processes [18]. 

In the case of siRNA, it has also been reported that a close relationship exists between the efficiency of siRNA release and the knockdown effect [1,2,3,7]. Kissel and coworkers succeeded (using PEI-graft-polyethyleneglycol (PEG)) in improving siRNA release to enhance the siRNA effect, by decreasing the extent of electrostatic interactions between polycations and siRNA [7]. On the other hand, we demonstrated that different mechanisms are operative for nanoparticle formation between pDNA and siRNA using a polycation, suggesting the manner in which pDNA and siRNA are largely different [19]. Based on these reports, a study of the relationship between nanoparticle formation of siRNA and the siRNA knockdown effect can be important issue. 

We previously reported on an optimal mono-cationic detergent (MCD), a quaternary ammonium salt detergent, for use as a pDNA condensation agent that results in efficient release, and showed a high transfection activity in cultured HeLa cells [20]. Moreover, the results of MCD screening showed that the hydrophobic nature of the MCD is important for the condensation of pDNA. In this study, we investigated the issue of whether a different extent of hydrophobic interaction could affect the formation of siRNA/MCD nanoparticles using various types of MCD and gene silencing efficiency to determine whether an MCD variant could be useful as a siRNA complexation agent. We first prepared siRNA nanoparticles using various MCDs with different lengths of alkyl moiety, and measured their diameters and zeta-potentials to investigate how the magnitude of the hydrophobic interaction can affect the formation of siRNA/MCD nanoparticles. We next packaged the siRNA/MCD nanoparticles into a lipid bi-layer equipped with octaarginine (R8-MEND, an octaarginine-modified multifunctional envelope-type nano device) system [21,22,23], which was previously reported to function as a non-viral gene delivey system similar to that of adenovirus vector [24,25]. The gene silencing efficiencies between the siRNA/MCD nanoparticles and Lipofectamine 2000 were then compared using HeLa cells expressing the Luciferase protein. Finally, we investigated the effect of siRNA nanoparticles formed with various types of MCDs on gene silencing efficiency. 

## 2. Results and Discussion

### 2.1. Preparation of siRNA/MCD Complex

We first prepared siRNA nanoparticles that contained various MCDs, and investigated the relationship between the N/P ratio and the diameter or zeta-potential of siRNA/MCD nanoparticles. In this experiment, we used various MCDs, which are shown in Table 1 (see [20] for details). 

**Table 1:** Structures of the mono-cationic detergents (MCD).

Group	Name	Basic structure	R (L-chain)
Type-C	C-1	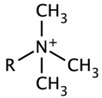	-C_14_H_25_
	C-2		-C_16_H_33_
Type-D	D-1	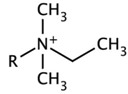	-C_12_H_25_
	D-2		-C_16_H_33_
Type-E	E-1	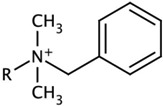	-C_12_H_25_
	E-2		-C_14_H_29_
	E-3		-C_16_H_33_

**Figure 1:**
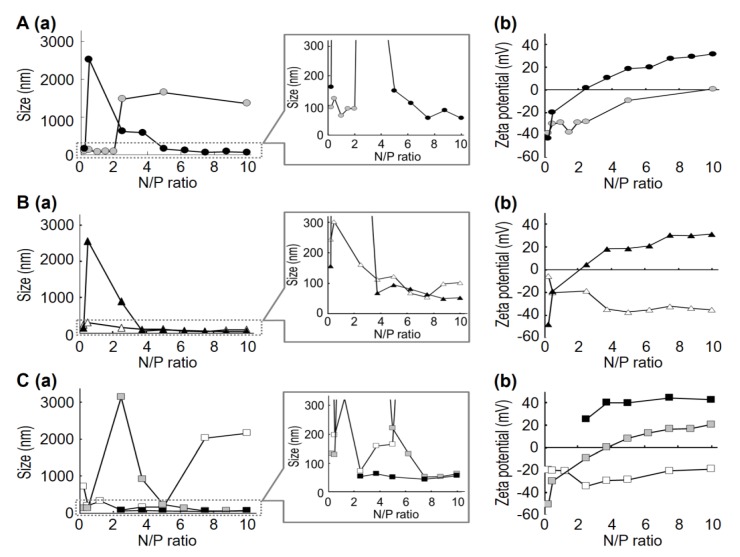
Relationship between the N/P ratio and diameter or zeta-potential of siRNA/MCD nanoparticles prepared using various MCDs. In this experiment, siRNA targeting GFP was used for preparing the nanoparticles. (**A**): the diameters (**a**) and zeta-potentials (**b**) of siRNA/MCD nanoparticles formed using Type-C in a series of N/P ratios: gray circles, C-1; closed circles, C-2; (**B**): the diameters (**a**) and zeta-potentials (**b**) of siRNA/MCD nanoparticles formed using Type-D at a series of N/P ratios: open triangles, D-1; closed triangles, D-2; (**C**): the diameters (**a**) and zeta-potentials (**b**) of siRNA/MCD nanoparticles formed using Type-E in a series of N/P ratios: open squares, E-1; gray squares, E-2; closed squares, E-3. Data are the means (n = 1–3).

The basic MCD structure is a quaternary ammonium compound containing two methyl groups. Type-C and -D MCDs have one short aliphatic chain (S-chain) and one long aliphatic chain (L-chain). Type-E MCDs contain one L-chain and one aromatic group. Physicochemical properties of the siRNA/MCD nanoparticles are summarized in Figure 1 and Table S1. Figure 1A,B show the diameters and zeta potentials for siRNA/MCD nanoparticles formed using type-C and type-D MCDs. When MCDs containing a hexadecyl group (-C_16_H_33_) L-chain were used (C-2 and D-2), positively charged nanoparticles were formed at high N/P ratios (Figure 1A, closed circles (C-2); Figure 1B, closed triangles (D-2)). In contrast, MCDs that have an L-chain of 12 carbons in length (D-1) formed negatively charged nanoparticles at high N/P ratios (Figure 1B, open triangles). It is presumed that siRNA/MCD nanoparticles would be formed by an assembly of MCD and siRNA via electrostatic and hydrophobic interactions. In the case of MCD containing long L-chains (C-2 and D-2), the long L-chain of MCD could bind to the hydrophobic region of siRNA to support the formation of siRNA/MCD nanoparticles. Thus, several cationic groups of MCD would be displayed in the surface of siRNA/MCD nanoparticles, resulting in the formation of positively charged nanoparticles. In the case of MCD containing short L-chains (D-1), the cationic group of MCD would largely contribute to siRNA/MCD nanoparticle formation. Thus, siRNA would be displayed in the surface of the siRNA/MCD nanoparticle, resulting in the formation of negatively charged nanoparticles, even at high N/P ratios. When MCDs containing a tetradecyl group (-C_14_H_29_) L-chain were used (C-1), aggregation was observed at high N/P ratios (Figure 1A, gray circles). In this situation, both the cationic group of MCD and the anionic region in siRNA may not be positions to permit them to be displayed in the surface of nanoparticles, resulting that the overall charge on the particle becoming neutral. We considered that the hydrophobic property of the nanoparticles promoted the assembly of nanoparticles via hydrophobic interactions to induce aggregation.

This trend also was observed when siRNA/MCD nanoparticles were prepared using MCDs containing an aromatic group (Type E MCDs), although MCDs that have L-chain lengths of 14 carbons (E2) resulted in the formation of positively charged nanoparticles at high N/P ratios (Figure 1C, gray squares). We considered that Type-E MCDs, which have an L-chain and an aromatic group, may form siRNA/MCD nanoparticles more effectively than Type-C and -D MCDs, due to the greater hydrophobicity of Type-E MCDs. A similar tendency was also observed when pDNA was used [20]. In the case of MCDs containing a dodecyl group L-chain (E-1), aggregation was observed at high N/P ratios, although negatively charged nanoparticles were formed at low N/P ratios. Collectively, the use of an MCD molecule with a long L-chain resulted in the formation of positively charged nanoparticle at high N/P ratios. In contrast, negatively charged nanoparticles were formed at low N/P ratios when an MCD with a short L-chain was used. These results suggest that the length of the L-chain, which largely contributes to the hydrophobicity of MCD, plays an important role in siRNA/MCD nanoparticles formation.

### 2.2. Comparison of Gene Silencing Efficiencies and Cell Viabilities Between siRNA/MCD Nanoparticles and LFN 2000

**Figure 2:**
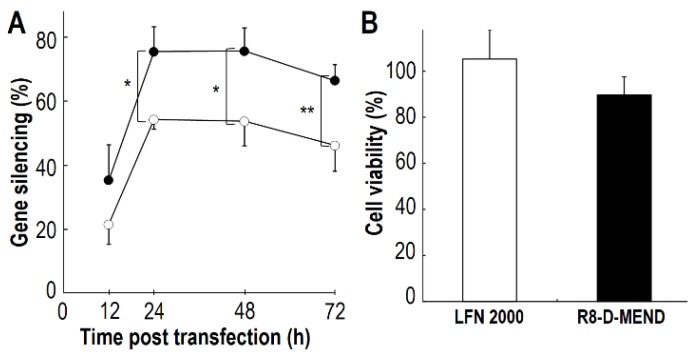
Evaluation of the gene silencing efficiency and cell viability. Gene silencing efficiencies and cell viability were evaluated, when 0.1 μg of siRNA was transfected into HeLa cells stably expressing luciferase. (**A**) Gene silencing efficiencies of R8-D-MEND (E3) (closed circles) and Lipofectamine 2000 (open circles) were evaluated at 12, 24, 48 and 72 h after transfection. Significant differences between the R8-D-MEND (E3) and Lipofectamine 2000 were determined by two-tail unpaired student’s t-test (** *p* < 0.01 * *p* < 0.05). Data are means ± S.D. (n = 3–4). (**B**), Cell viabilities of R8-D-MEND (E3) (closed bar) and Lipofectamine 2000 (open bar) were evaluated at 48 h after transfection. No significant differences between the R8-D-MEND (E3) and Lipofectamine 2000 were found when the two-tail unpaired student’s t-test was applied. Data are the means ± S.D. (n = 3–4).

To determine whether siRNA/MCD nanoparticles exhibit significant gene silencing, the cytosolic delivery of siRNA/MCD nanoparticles was examined using the R8-MEND system and the gene silencing efficiency was evaluated using HeLa cells expressing the Luciferase protein. In this experiment, we prepared the R8-D-MEND containing an siRNA nanoparticle complexed with the E-3 MCD, which showed high transfection activity as a DNA condenser [20]. Gene silencing efficiencies after 12, 24, 48 and 72 h of transfection are shown in Figure 2A. In the case of the R8-D-MEND (E-3), the gene silencing efficiency was around 40% at 12 h after transfection and approached 80% at 24 h after transfection. The gene silencing of luciferase gene expression was maintained up to 48 h after transfection (Figure 2A, closed circles). We also found that the R8-D-MEND (E-3) showed a significantly higher gene silencing efficiency than Lipofectamine 2000 at 24–72 h after transfection (Figure 2A). We also investigated the cell viability based on protein content. As a result, no significant differences of cell viabilities between R8-D-MEND (E-3) and LFN2000 were observed (Figure 2B). We concluded that the R8-D-MEND (MCD) showed a higher gene silencing efficiency than Lipofectamine 2000, and the transfection could be safely performed.

### 2.3. Investigation of siRNA Nanoparticle Formed with Various Type of MCD on Gene Silencing Efficiencies

**Figure 3:**
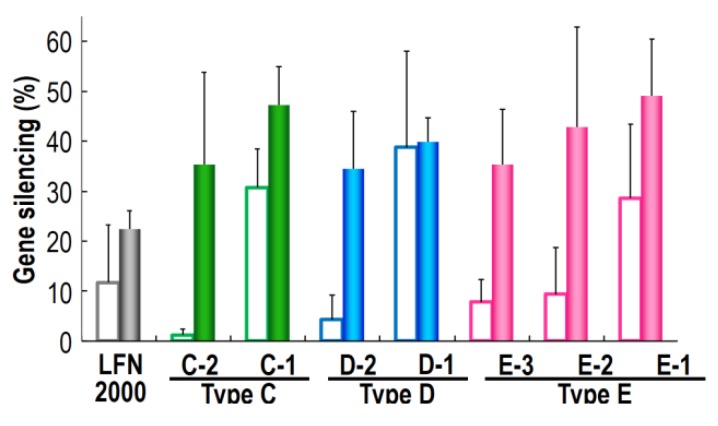
Investigation of the relationship between the hydrophobicity of the MCD and the gene silencing efficiency of the R8-D-MEND (MCD). The gene silencing efficiencies at 12 h after transfection were evaluated as described in Materials and Methods. Closed bars, 0.1 μg of siRNA; open bars, 0.04 μg of siRNA. Data are means ± S.D. (n = 3).

We investigated the gene silencing efficiency of siRNA nanoparticles formed with various type of MCDs, using the R8-D-MEND (MCD). In this experiment, we prepared an R8-D-MEND containing siRNA nanoparticles complexed with various MCDs. Since the cellular uptake of the carriers depends on the R8 modified to the envelope of the carrier, as previously reported [22], it was predicted that all of the formulations would be internalized to the same extent. We previously determined the lipid composition, which included PA (DOPE/PA/STR-R8 (7:2:1, molar ratio)) required for achieving an optimal endosome-fusogenic composition [26,27]. The lipid composition of the envelopes took this into consideration. We measured the sizes and zeta potential of the R8-D-MEND (MCD) for a range of N/P ratios (data not shown). We chose specific N/P ratio of each MCD to form R8-D-MEND (MCD) without aggregation. The characteristics of the siRNA/MCD nanoparticles and the R8-D-MEND (MCD) are summarized in Table 2. Figure 3 provides information on the gene silencing efficiency of various R8-D-MEND (MCD) at 12 h after transfection when 0.1 μg and 0.04 μg of siRNA was transfected to HeLa cells expressing Luciferase protein. In each MCD group, the increase in the gene silencing efficiency was proportional to the decrease in the length of the L-chain in the MCD. The results suggest that the release of siRNA from siRNA nanoparticles complexed with MCD containing a long L-chain may not be effective, because the MCDs may form tight siRNA/MCD nanoparticles, depending on the extent of increase in the length of L-chains. It was not possible to confirm this hypothesis, because it was not possible to precisely determine the release efficiency of siRNA from each siRNA/MCD. This issue is a subject for being investigated in the future. We also found that siRNA/MCD nanoparticles with negative charge showed a higher gene silencing than siRNA/MCD nanoparticles with a positive charge. The results suggest that siRNA/MCD nanoparticles with a positive charge may interact siRNA released from a siRNA/MCD nanoparticle in the cytosol to inhibit gene silencing. 

**Table 2:** Characteristics of nanoparticles of siRNA targeting Luciferase formed using various MCDs Data are represented by the mean ± S.D. (n = 3–6).

MCD (N/P ratio)	siRNA/MCD nanoparticle			R8-D-MEND		
	Size (nm)	Zeta potential (mV)	PDI	Size (nm)	Zeta potential (mV)	PDI
C-1 (1.0)	130 ± 65	−15.5 ± 19.3	0.48 ± 0.08	111 ± 9	32.4 ± 3.6	0.22 ± 0.04
C-2 (5.0)	131 ± 60	13.1 ± 5.6	0.35 ± 0.11	110 ± 3	43.3 ± 3.1	0.24 ± 0.06
D-1 (5.0)	51 ± 18	−13.1 ± 7.7	0.32 ± 0.15	92 ± 13	30.3 ± 13.4	0.20 ± 0.03
D-2 (5.0)	85 ± 27	10.2 ± 3.9	0.25 ± 0.02	123 ± 10	45.0 ± 3.6	0.18 ± 0.03
E-1 (2.5)	85 ± 26	−34.6 ± 11.4	0.28 ± 0.08	105 ± 9	30.9 ± 6.4	0.20 ± 0.05
E-2 (7.5)	60 ± 8	4.9 ± 3.4	0.24 ± 0.03	109 ± 11	43.7 ± 0.8	0.20 ± 0.03
E-3 (5.0)	67 ± 7	32.6 ± 2.5	0.33 ± 0.06	113 ± 7	51.0 ± 2.5	0.26 ± 0.02

**Figure S1:**
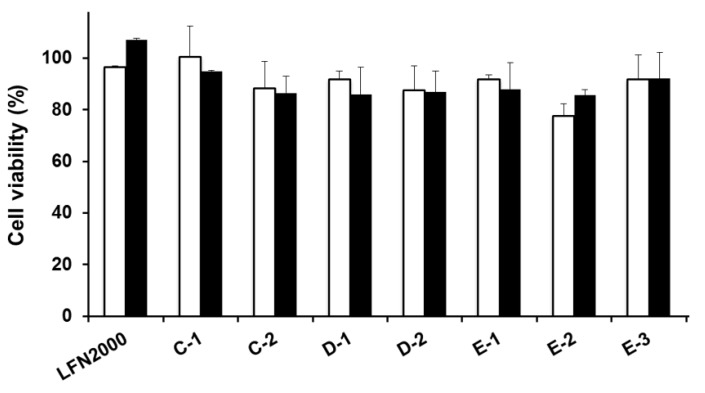
Cell viabilities of R8-D-MEND and Lipofectamine 2000 were evaluated at 24 h after transfection, using siRNA targeting Luciferase (closed bar) and siRNA targeting GFP (open bar). No Significant differences between the siRNA targeting Luciferase and siRNA targeting GFP were determined by the two-tail unpaired student’s t-test. Data are represented the means ± S.D. (n = 3–4).

We also investigated cell viability based on protein content, when siRNA targeting Luciferase and siRNA targeting GFP were transfected into HeLa cells using R8-D-MEND, respectively (Figure S1). As a result, no significant differences in cell viability were found between siRNA targeting Luciferase and siRNA targeting GFP in each MCD. The results suggest that the R8-D-MEND (MCD) would rule out toxicity as a factor in the decrease in luciferase expression.

## 3. Experimental

### 3.1. Materials

The anti-Luciferase siRNA (21mer, Antisense sequence: 5'-GGGUUG GCACCAGCAGCGCTT-3', Sense sequence: 5'-GCGCUGCUGGUGCCAACCCTT-3'), anti-green fluorescent protein (GFP) siRNA (21mer, Antisense sequence: 5'-GAUGAACUUCAGGGUCAGCTT-3', Sense sequence: 5'-GCUGACCCUGAAGUUCAUCTT-3') were purchased from Hokkaido System Science Co., Ltd (Hokkaido, Japan). Diethylpyrocarbonate (DEPC)-treated water was purchased from Nacalai Tesque, Inc (Kyoto, Japan). Trimethyltetradecylammonium bromide (C-1), cetyltrimethylammonium bromide (C-2), dodecylethyldimethylammonium bromide (D-1), ethylhexadecyldimethylammonium bromide (D-2), benzyldodecyldimethylammonium bromide (E-1), benzyldimethyltetradecylammonium chloride (E-2) and benzyldimethylhexadecylammonium chloride (E-3) were purchased from Sigma-Aldrich (St. Louis, CA, USA), as purified form. 1, 2-Dioleoyl-sn-glycero-3-phosphoethanolamine (DOPE) was purchased from AVANTI Polar Lipids Inc. (Alabaster, AL, USA). Phosphatidic acid (PA) was purchased from Sigma-Aldrich. Stearyl octaarginine (STR-R8) was purchased from Kurabo Industries Ltd. (Osaka, Japan). Dulbecco’s modified Eagle medium (DMEM), Opti-MEM, and Lipofectamine 2000 Reagent (LFN2000) were purchased from Invitrogen Corp. (Carlsbad, CA, USA). Fetal bovine serum was purchased from HyClone (South Logan, UT, USA). G418 disulfate was purchased from Nacalai Tesque, Inc. All other chemicals used were commercially available reagent-grade products.

### 3.2. Preparation of siRNA/MCD Nanoparticle

1 M MCD stock solutions were prepared by dissolving various MCDs in EtOH. The MCD stock solutions were diluted to the optimal concentration with DEPC-treated water before use. siRNA dissolved in DEPC-treated water (0.1 mg/mL) was mixed with an equal volume of MCD solution to form siRNA/MCD nanoparticles by vortexing at room temperature. siRNA/MCD nanoparticles were formed using various MCDs in a series of nitrogen/phosphate (N/P) ratios.

### 3.3. Measurement of Size and Zeta-Potential of Carriers

Particle sizes of siRNA/MCD nanoparticle (0.05 mg/mL siRNA concentration) and R8-D-MEND (0.55 mM lipid concentration) were measured using a quasi-elastic light scattering method. The zeta-potentials of siRNA/MCD nanoparticle (0.01 mg/mL siRNA concentration) and R8-D-MEND (0.1 mM lipid concentration) were determined electrophoretically by means of an electrophoretic light scattering spectrophotometer (Zetasizer Nano ZS; Malvern Instruments, Malvern, Worcestershire, UK). The samples were evaluated at 25 °C.

### 3.4. Construction of R8-D-MEND Containing siRNA/MCD Nanoparticle

A lipid film was prepared by the evaporation of a chloroform solution of 1.1 µmol lipid (DOPE/PA = 7:2 (molar ratio)) on the bottom of a glass tube, followed by hydration with 2 mL of DEPC-treated water for 15 min at room temperature. The glass tube was sonicated in a bath-type sonicator (AU-25C; Aiwa Co., Tokyo, Japan), followed by sonication for 10 min in ice-cold water with a probe-type sonicator (Digital Sonifier 250; Branson Ultrasonics Co., Danbury, CT, USA) to produce small unilamellar vesicle (SUV). To prepare octaarginine-modified SUV (R8-SUV), a solution of STR-R8 (10 mol% of total lipids) was added to an SUV suspension and the resulting suspension was then incubated for 30 min at room temperature. The physicochemical properties of the materials are summarize in Table S2. siRNA/MCD nanoparticles (final siRNA concentration, 0.05 mg/mL) were prepared at optimized N/P ratio of 1.0 (C-1), 5.0 (C-2), 5.0 (D-1), 5.0 (D-2), 2.5 (E-1), 7.5 (E-2), 5.0 (E-3), respectively. In the case of positively charged siRNA nanoparticles complexed with C-2, D-2 and E-3 MCDs, the R8-D-MEND was constructed by mixing siRNA/MCD nanoparticles with twice the volume of negatively charged SUV suspension, followed by incubation with STR-R8 (10 mol% of total lipids) for 30 min at room temperature. When the other MCDs were used to prepare siRNA/MCD nanoparticle, R8-SUV was used to prepare R8-D-MEND.

### 3.5. Evaluation of Gene Silencing Efficiency and Cell Viability

HeLa cells stably expressing luciferase (HeLa-Luc) (4.0 × 10^4^ cells) [1] were incubated in DMEM containing 400 µg/mL G418 and 10% fetal bovine serum under 5% CO_2_/air at 37 °C for 24 h. R8-D-MEND (MCD) containing 0.04, or 0.1 µg of siRNA-suspended in 0.25 mL of serum-free DMEM were added to the cells, followed by incubation under an atmosphere of 5% CO_2_/air at 37 °C for 3 h. LFN2000, as a control, was used according to the manufacturer’s protocol. siRNA/LFN2000 complex-suspended in 0.25 mL of serum-free Opti-MEM were added to the cells, followed by incubation under an atmosphere of 5% CO_2_/air at 37 °C for 3 h. After washing the cells with phosphate-buffer saline (PBS, 137 mM NaCl, 2.68 mM KCl, 8.05 mM, Na_2_HPO_4_, 1.47 mM KH_2_PO_4_, pH 7.4), 1 mL of fresh DMEM containing 10% serum was added to the cells, followed by incubation for 9, 21, 45, or 69 h. The cells were then washed, and luciferase activity was measured using a Luciferase Assay System with a Reporter Lysis Buffer kit (Promega; Madison, WI, USA) by means of a luminometer (Luminescencer-PSN; ATTO, Japan). Cellular protein content was determined using a BCA protein assay kit (PIERCE; Rockford, IL, USA) and microplate reader (Benchmark Plus; Bio-Rad laboratories, Inc, Hercules, CA, USA). 

Gene silencing efficiency was calculated as follows:

Gene silencing efficiency (%) = (1 − L_Luc_/L_GFP_) × 100

where L_Luc_, L_GFP_ represent the luciferase activity when samples which containing anti-Luc and anti-GFP siRNAs were transfected, respectively. 

Cell viability was calculated as follows:

Cell viability (%) = C_S_/C_N_ × 100

where C_S_, C_N_ represent the cellular protein concentration when cells were treated with samples containing of siRNA and with naked siRNA, respectively.

### 3.6. Statistical Analysis

The diameter, zeta-potentials and polydispersity index (PDI) of siRNA/MCD nanoparticles formed using various MCDs at a series of N/P ratios were measured from one to three times respectively. Each value shown in Figure 1 and Table S1 is represented by the mean (n = 1−3). Gene silencing efficiency and cell viability compared between R8-D-MEND (E3) and LFN2000 were evaluated three to four times. Each value shown in Figure 2 is represented by the mean ± S.D. (n = 3−4). Statistical significances between the R8-D-MEND (E3) and Lipofectamine 2000 were examined by the by two-tail unpaired student’s t-test. Levels of *p* < 0.05 were considered to be significant. The construction of each R8-D-MEND were repeated three to six times respectively. Each value shown in Table 2 is represented by the mean ± S.D. (n = 3−6). Gene silencing efficiencies of each R8-D-MEND were investigated three times respectively. Each value shown in Figure 3 is represented by the mean ± S.D. (n = 3).

## 4. Conclusions

The formation of siRNA nanoparticles using various MCDs was investigated, and the findings indicate that an MCD with a long L-chain formed positively charged nanoparticles. It is presumed that the long L-chain of MCD binds to the hydrophobic region of siRNA to support the formation of siRNA/MCD nanoparticles, in which several cationic groups of MCD would be displayed on the surface of the nanoparticles. In contrast, negatively charged nanoparticles were formed when MCD with a short L-chain was used. In this case, the cationic group of the MCD would largely contribute to siRNA/MCD nanoparticle formation, resulting in the siRNA displayed on the surface of the nanoparticle. We also confirmed that the siRNA/MCD nanoparticles showed a higher gene silencing efficiency than Lipofectamine 2000. Moreover, the increase in the gene silencing efficiency was proportional to the decrease in the length of L-chain in MCD. We also found that siRNA/MCD nanoparticles with a negative charge showed a higher gene silencing than siRNA/MCD nanoparticles with a positive charge. Such information provides an alternate viewpoint for design of siRNA vectors.

## Supplementary Materials

**Table S1:** Characteristics of nanoparticles of siRNA targeting GFP prepared using various MCDs at a range of N/P ratios.

Nanoparticles prepared using C-1				Nanoparticles prepared using C-2			
N/P ratio	Size (nm)	Zeta potential (mV)	PDI	N/P ratio	Size (nm)	Zeta potential (mV)	PDI
0.25	94	−37.9	0.72	0.25	162	−42.9	0.63
0.5	124	−29.8	0.55	0.5	2520	−19.9	0.37
1	66	−28.8	0.33	2.5	616	1.6	0.6
1.5	90	−37.5	0.36	3.75	584	10.7	0.57
2	90	−28.6	0.26	5	151	18.7	0.43
2.5	1480	−28.1	0.58	6.25	108	19.9	0.27
5	1650	−9.3	0.39	7.5	58	27.7	0.37
10	1360	0.8	0.56	8.75	84	29.3	0.42
				10	57	31.7	0.36
**Nanoparticles prepared using D-1**				**Nanoparticles prepared using D-2**			
**N/P ratio**	**Size (nm)**	**Zeta potential (mV)**	**PDI**	**N/P ratio**	**Size (nm)**	**Zeta potential (mV)**	**PDI**
0.25	244	−5.6	0.67	0.25	156	−47.8	0.44
0.5	302	−19.9	0.35	0.5	2560	−18.6	0.7
2.5	162	−18.5	0.34	2.5	888	4.5	0.62
3.75	113	−34.4	0.34	3.75	69	18.3	0.24
5	123	−37.0	0.22	5	95	18.7	0.32
6.25	69	−35.0	0.3	6.25	82	21	0.34
7.5	55	−31.9	0.21	7.5	65	30.4	0.36
8.75	99	−33.5	0.24	8.75	50	30	0.34
10	102	−35.0	0.25	10	53	31.3	0.36
**Nanoparticles prepared using E-1**				**Nanoparticles prepared using E-2**			
**N/P ratio**	**Size (nm)**	**Zeta potential (mV)**	**PDI**	**N/P ratio**	**Size (nm)**	**Zeta potential (mV)**	**PDI**
0.25	713	−19.9	0.85	0.25	133	−50.2	0.41
0.5	198	−20.0	0.49	0.5	129	−29.6	0.24
1.25	324	−20.7	0.66	2.5	3140	−9.3	0.455
2.5	74	−34.2	0.28	3.75	918	0.3	0.45
3.75	159	−29.1	0.18	5	221	8.2	0.386
5	165	−28.5	0.14	6.25	132	12.9	0.22
7.5	2030	−20.5	0.7	7.5	53	16.5	0.26
10	2160	−18.8	0.6	8.75	54	16.9	0.28
				10	63	20.5	0.313
**Nanoparticles prepared using E-3**							
**N/P ratio**	**Size (nm)**	**Zeta potential (mV)**	**PDI**				
2.5	54	25.2	0.27				
3.75	63	40.1	0.25				
5	53	39.7	0.36				
7.5	45	44.4	0.32				
10	57	42.8	0.42				

Data are represented by the mean (n = 1–3).

**Table S2:** Characteristics of SUV and R8-SUV.

	Size (nm)	Zata potential (mV)
SUV	74 ± 10	−66.8 ± 8.9
R8-SUV	86 ± 14	29.9 ± 11.3

Data are represented by the mean ± S.D. (n = 6).
